# Psychotic Disorder Secondary to Cerebral Venous Thrombosis Caused by Primary Thrombophilia in a Pediatric Patient with Protein S Deficiency and an MTHFR p.Ala222Val Variant: A Case Report

**DOI:** 10.3390/hematolrep17040034

**Published:** 2025-07-03

**Authors:** Darío Martínez-Pascual, Alejandra Dennise Solis-Mendoza, Jacqueline Calderon-García, Bettina Sommer, Eduardo Calixto, María E. Martinez-Enriquez, Arnoldo Aquino-Gálvez, Hector Solis-Chagoyan, Luis M. Montaño, Bianca S. Romero-Martinez, Ruth Jaimez, Edgar Flores-Soto

**Affiliations:** 1 Escuela de Posgrado en Sanidad Naval, Universidad Naval, Secretaría de Marina de México, Veracruz 94077, Mexico; dario.mtz_21@hotmail.com (D.M.-P.); denisesolis88@gmail.com (A.D.S.-M.); 2 Departamento de Hematología, Hospital Naval de Especialidades de Veracruz, Veracruz 91918, Mexico; dra.jacqueline.cg@gmail.com; 3 Departamento de Investigación en Hiperreactividad Bronquial, Instituto Nacional de Enfermedades Respiratorias “Ismael Cosío Villegas”, Mexico City 14370, Mexico; bsommer195@gmail.com; 4 Laboratorio de Neurofarmacología, Subdirección de Investigaciones Clínicas, Instituto Nacional de Psiquiatría Ramón de la Fuente Muñiz, Mexico City 14370, Mexico; neurolalocalixto@yahoo.com.mx; 5 Departamento de Farmacología, Facultad de Medicina, Universidad Nacional Autónoma de México, Mexico City 04510, Mexico; emartinez@facmed.unam.mx (M.E.M.-E.); lmmr@unam.mx (L.M.M.); biancasromero_@hotmail.com (B.S.R.-M.); 6 Laboratorio de Biología Molecular, Departamento de Fibrosis Pulmonar, Instituto, Instituto Nacional de Enfermedades Respiratorias Ismael Cosío Villegas, Mexico City 14080, Mexico; araquiga@yahoo.com.mx; 7 Laboratorio de Neurobiología Cognitiva, Centro de Investigación en Ciencias Cognitivas, Universidad Autónoma del Estado de Morelos, Cuernavaca 62209, Mexico; hecsolcha@yahoo.com.mx

**Keywords:** psychotic disorder, primary thrombophilia, MTHFR, protein S

## Abstract

**Background and Clinical Significance:** Herein, we describe the clinical case of a 17-year-old patient with psychotic disorder secondary to cerebral venous thrombosis due to primary thrombophilia, which was related to protein S deficiency and a heterozygous MTHFR gene mutation with the p.Ala222Val variant. **Case presentation:** A 17-year-old female, with no history of previous illnesses, was admitted to the emergency service department due to a psychotic break. Psychiatric evaluation detected disorganized thought, euphoria, ideas that were fleeting and loosely associated, psychomotor excitement, and deviant judgment. On the fifth day, an inflammatory process in the parotid gland was detected, pointing out a probable viral meningoencephalitis, prompting antiviral and antimicrobial treatment. One week after antiviral and steroidal anti-inflammatory treatments, the symptoms’ improvement was minimal, which led to further neurological workup. MRI venography revealed a filling defect in the transverse sinus, consistent with cerebral venous thrombosis. Consequently, anticoagulation treatment with enoxaparin was initiated. The patient’s behavior improved, revealing that the encephalopathic symptoms were secondary to thrombosis of the venous sinus. Hematological studies indicated the cause of the venous sinus thrombosis was a primary thrombophilia caused by a heterozygous MTHFR mutation variant p.Ala222Val and a 35% decrease in plasmatic protein S. **Conclusions:** This case highlights the possible relationship between psychiatric and thrombotic disorders, suggesting that both the MTHFR mutation and protein S deficiency could lead to psychotic disorders. Early detection of thrombotic risk factors in early-onset psychiatric disorders is essential for the comprehensive management of patients.

## 1. Introduction

Thrombophilia is a multifactorial blood alteration characterized by a predisposition to develop thrombosis in veins and/or arteries, originating from genetic, environmental, and acquired conditions that determine its clinical expression [1]. It has been classified into two types: primary or inherited and secondary. Among the most common causes of primary thrombophilia, mutations in the methylenetetrahydrofolate reductases (MTHFR) enzyme, protein S (PS) and protein C (PC) deficiency, presence of lupus anticoagulant/antiphospholipid syndrome, mutations that increase risk of a hypercoagulable state like Factor V Leiden (FVL), and mutations of the prothrombin gene have been reported [2]. MTHFR deficiency is a rare disorder of folate and sulfide-containing amino acid metabolism, characterized by low plasma folate levels, hyperhomocysteinemia, hypomethioninemia, and lack of methylmalonic aciduria. The most prevalent mutation in the MTHFR gene is C677T; although widespread, it can vary between ethnic and regional groups. In the U.S., approximately 20–40% of white and Hispanic individuals carry one copy of the C677T mutation (heterozygous). On the other hand, it is only seen in 1–2% of Black individuals. Homozygous mutations (individuals carrying two copies) range between 8–20% of people in North America, Europe, and Australia. The heterozygous mutation C677T typically has about 65% of the normal MTHFR enzyme activity, while homozygous individuals show a more significant reduction, with only about 30% of the normal function [3].

Another variant, known as A1298C, is present in about 7% to 12% of individuals in North America, Europe, and Australia. It is less frequent among Hispanics (4–5%) and among Chinese and other Asian populations (1–4%). Homozygosity for A1298C results in around 60% of normal enzyme activity. Some individuals may carry one mutated copy of both C677T and A1298C, a condition referred to as compound or double heterozygosity, which can also lead to reduced MTHFR enzyme function.

Clinically, it often presents during the neonatal period or in childhood, rarely in adolescence or adulthood, and may present with neurological symptoms such as encephalopathy, psychomotor retardation, gait disturbances, and epilepsy. These symptoms and clinical changes may be associated with thrombotic events [4]. On the other hand, PS is a multifunctional protein that acts in blood coagulation, inflammation, and other cellular processes. Its plasma concentration is about 350 nM, 60% of which is bound to complement protein 4 (C4BP), and the other 40% is free, with both forms performing anticoagulant functions [5]. PS deficiency in the newborn can cause severe thrombosis and alterations in the development of the vascular system, with fatal consequences. In this sense, the anticoagulant effects of PC (or PS) are exercised through its qualities as a cofactor of activated PC (APC), cofactor of the tissue factor pathway inhibitor (TFPI), and its inhibition of the factor IXa function [5]. Besides, the mutations in the MTHFR gene and protein S deficiency in cases of severe mental disorders have been reported; these mutations can lead to neurometabolic diseases. Conceivably, an alteration in a metabolic process might lead to the deficiency of an essential product or the accumulation of some harmful substance, causing a significant impact on the different systems involved, and this circumstance can manifest itself acutely with a myriad of symptoms, such as digestive and respiratory disorders, neurological deterioration, psychiatric symptoms, or behavioral alterations. However, the identification of neuropsychiatric disorders related to a metabolic background, especially in pediatric patients, remains difficult, even though the joint presentation with other alterations can guide the diagnosis [6]. In this sense, a psychotic disorder is characterized by a distorted sense of reality, along with disruptions in thinking, perception, and behavior, and/or hallucinations that occur in various neuropsychiatric and medical conditions [7,8]. It has been reported that expression of MTHFR gene polymorphisms may be a risk factor for psychotic disorder, bipolar disorder, and schizophrenia, particularly the C/T transition at nucleotide 677 in exon 4, which produces the amino acid substitution from alanine to valine (Ala222Val). In homozygotes with the C677T polymorphism, only 30% of the MTHFR enzymatic activity remains, while in heterozygotes, 65% of its activity is retained [7,9]. Moreover, the MTHFR gene is linked with other at-risk genes, such as the Val66Met mutation of the brain-derived neurotrophic factor (BDNF), which can interact in patients with a first psychotic episode, as seen in the effects of the hippocampal volume [10]. On the other hand, PS deficiency has also been found in patients with psychiatric disorders. A study comparing 70 schizophrenic patients with 98 controls showed a deficiency of free protein S in 22% of the patients, but this was not detected in any control [9]. Furthermore, free PS deficiency is associated with a 145-fold increase in the risk of having a first-degree relative with schizophrenia [9]. The present paper seeks to illustrate how genetic mutations linked to primary thrombophilia and decreased plasma PS concentrations may contribute to the development of psychotic disorder in a 17-year-old patient, in order to identify specific therapeutic targets for a more precise approach and to broaden the knowledge about metabolically related psychiatric illnesses.

## 2. Case Presentation

**Figure 1:**
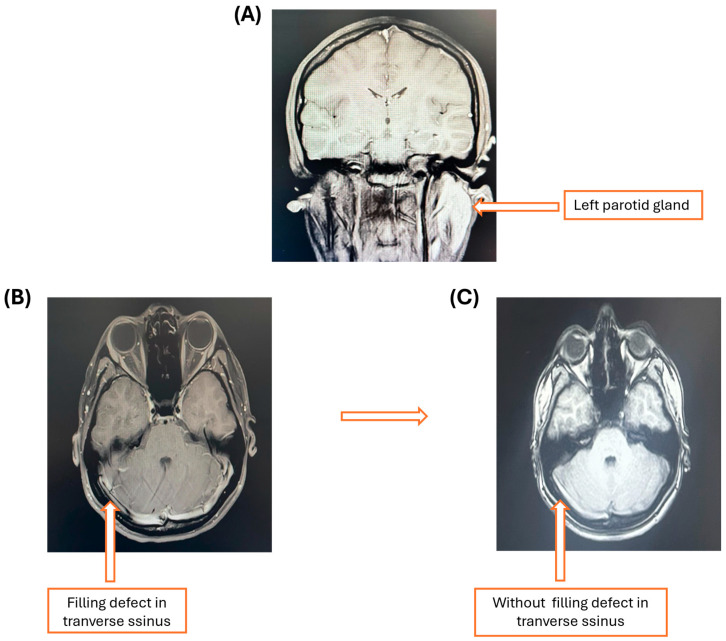
Contrast-enhanced magnetic resonance imaging of the brain. (**A**) Image showing left parotid gland enlargement. (**B**) Magnetic resonance venography with a filling defect of the transverse sinus and hyperintensity at the confluence of the sinuses can be appreciated. (**C**) Magnetic resonance venography post-treatment without filling defect.

A 17-year-old female of Afro-Mestizo ethnicity was admitted to the emergency room due to a psychotic outbreak less than 48 h after onset. She had had no previous psychiatric events, was conscious but disoriented, showed nonsensical speech, had fleeting and megalomaniacal ideas, disorganized judgment, and psychomotor agitation. Laboratory blood studies were normal, and cytochemical and cytological analysis of cerebrospinal fluid (CSF), magnetic resonance imaging with gadolinium contrast of the brain, and an electroencephalogram were requested. The initial diagnosis was psychosis with suspected autoimmune encephalitis. Lorazepam 2 mg IV every 6 h and olanzapine 10 mg/day IM were the initial treatment. The patient was evaluated by the staff of the psychiatry and neurology departments, and, on the fifth day, she continued with the same psychotic pattern, without showing signs of a systemic inflammatory response, but with notable inflammation in the left parotid gland (Figure 1); a viral panel was requested, and a positive cytomegalovirus result was obtained (Table 1).

Antiviral treatment was started with ganciclovir 5 mg/kg IV every 12 h, dexamethasone IV, 8 mg every 8 h; dexmedetomidine in 0.9% saline titrated from 0.5 to 1 mcg/kg/hour IV and, in case of agitation, olanzapine 5 mg every 12 h IV. The cytological and cytochemical CSF studies were within normal limits, while the electroencephalogram (EEG) showed neuronal activity related to encephalopathy without epileptic activity. One week after treatment initiation, the patient did not show significant improvement. A venous magnetic resonance imaging (MRI) was requested, and a filling defect in the transverse sinus was revealed, suggesting venous thrombosis of the transverse sinus (Figure 1), so anticoagulant therapy was started with enoxaparin 1 mg/kg/IV (60 mg). At this time, a history of a maternal grandmother with coagulopathy was documented. On the ninth day of admission, an improvement in behavior and neuropsychiatric symptoms secondary to the treatment of venous sinus thrombosis of undetermined origin began to be observed. On physical examination, confusion, disinhibition, and inattention persisted, with no signs of psychomotor excitement or catatonia, although with fluctuations in alertness.

Hematological studies were requested to rule out primary thrombophilia. Other studies were also carried out: a panel of mutations, quantification of anticoagulant proteins (protein C, protein S, and anti-thrombin), mutation 677c7 of the MTHFR gene, mutation of the prothrombin gene 20210a, and factor V Leiden mutation. The anticoagulant treatment was changed, and rivaroxaban 20 mg/day, orally, was given.

Finally, psychotic disorder secondary to cerebral venous thrombosis due to primary thrombophilia caused by an MTHFR gene variant and PS deficiency was diagnosed (Table 2). Laboratory findings revealed the presence of the heterozygous MTHFR gene with the p.Ala222Val mutation without reference value, as well as decreased levels of PS to 35% of its normal value (reference value: 54.0–103.0%). These values were taken 4 months after the initial presentation. Taken together, these findings supported the diagnosis: psychotic disorder attributed to venous thrombosis of the transverse sinus secondary to primary thrombophilia due to PS deficiency. Additionally, levels of homocysteine were measured, observing plasmatic levels of 36 µmol/L (reference value: <15 µmol/L).

The patient was discharged with the following therapeutic regimen: haloperidol 10 mg/day; magnesium valproate 200 mg every 8 h; rivaroxaban 20 mg/day; biperiden 2 mg every 8 h; esomeprazole 40 mg/day; the whole medication was orally administered.

## 3. Discussion

The present study reports for the first time a clinical case of the possible association between psychotic disorders and primary thrombophilia due to an MTHFR mutation and PS deficiency. In this sense, bipolar disorder with early onset during childhood or adolescence has been associated with a worse prognosis compared to cases with a later onset during adulthood and, in most cases, is linked to a genetic component [11]. Psychotic disorders are clinically characterized by hallucinations, delusions, and disorganized thinking and behavior, as observed in schizophrenia, although they can also occur in other psychiatric and medical conditions. Many children and adolescents report psychotic-like experiences, which may be related to other forms of psychopathology and personal history, such as trauma, substance use, or suicidal behavior [8]. The origin may be related to heredity, changes in the levels of some neurotransmitters, and maladaptation of psychological factors. On the other hand, neuropsychiatric disorders with a metabolic origin are difficult to identify, although the early signs and their association with extra-neurological manifestations such as hematological, gastrointestinal, or respiratory symptoms may also suggest a genetic component [6,8]. In the present case, the concomitant development of neuropsychiatric symptoms and thrombophilia suggested a neurometabolic alteration. Some of the signs that might guide clinical evaluation toward a neurometabolic disease are as follows: (1) resistance, refractory, or paradoxical response to antipsychotics; (2) serious neurological adverse effects after the administration of antipsychotics; (3) abrupt development of psychiatric disorders in patients with seizures or developmental delays; (4) early onset of psychiatric symptoms along with mental retardation or progressive cognitive impairment in children with prior normal development; and (5) presence of catatonia associated with atypical elements [6]. In the present case, the diagnosis was made based on the criteria from the DSM-5 [12]. The patient presented prominent hallucinations (Criterion A), which were evidently a consequence of the cerebral venous thrombosis secondary to primary thrombophilia, a fact confirmed by low plasmatic protein S levels and the presence of a homozygous MTHFR p.Ala222Val variant (Criterion B). The neurological imaging and clinical findings supported a direct pathophysiological link between the cerebral venous thrombosis and the onset of the psychotic symptoms. Furthermore, there was no evidence of a pre-existing psychiatric disorder that could better explain the symptomatology (Criterion C), and the symptoms were not limited to periods of fluctuating consciousness or delirium (Criterion D). Additionally, the psychotic symptoms significantly impaired the patient’s ability to function socially and academically, meeting Criterion E. In this context, this case meets all the diagnostic criteria for a psychotic disorder due to another medical condition, i.e., cerebral venous thrombosis due to primary thrombophilia associated with protein S deficiency and the MTHFR p.Ala222Val variant.

Mutations in MTHFR and the expression of PS have been widely documented in primary thrombophilia [1,2]. In this regard, the development of major psychiatric disorders such as depression and schizophrenia has been associated with an increase in thrombotic risk [9]. Interestingly, the mutation of the MTHFR gene has been associated with moderate to high levels of hyperhomocysteinemia [3]. In the present case, the patient showed plasmatic homocysteine levels of 36 µmol/L, considered to be moderately elevated, but nonetheless a risk factor for cardiovascular disease, including coronary disease, myocardial infarctions, and cardiovascular events [3]. Feasibly, healthy blood tissues ensure brain homeostasis, and any disturbance in them could unchain serious neuropsychiatric conditions. In this clinical case, acute steroid management and dosage cannot acutely generate bipolar disorder or changes associated with blood coagulation. Using drugs to reduce hypercoagulability significantly improved the patient’s behavior in the short term: neuronal metabolism (oxygenation, temperature, ATP levels, and neurochemistry) gradually recovered as flow and perfusion were restored, especially in the brain.

Besides its role in primary thrombophilia, MTHFR gene mutations have also been related to bipolar disorder and schizophrenia [7,8,9,10,11,13,14,15,16,17,18,19]. Likewise, free PS deficiency has been reported in patients with schizophrenia [9], although an association with bipolar disorder has not been established. The coexistence of neurological symptoms, such as epilepsy, along with thrombosis due to MTHFR deficiency has already been described in two brothers [4]. However, a case of bipolar disorder associated with thrombophilia in the same patient has not been reported yet. Considering the low prevalence of this mutation in association with thrombophilia, there are currently no indications to routinely screen for the MTHFR mutation in clinical guidelines. As stated by the American College of Obstetricians and Gynecologists (ACOG), the determination of homocysteine levels and polymorphisms for the MTHFR gene should be part of the diagnostic evaluation for venous thromboembolism or in cases of spontaneous abortion due to the lack of sufficient evidence to justify the indication [20]. Similarly, the British Committee for Standards in Haematology (BCSH) does not include testing for the MTHFR mutation in the standard evaluation of hereditary thrombophilia, since no clinical utility has been assessed for the inclusion of this test [21]. Furthermore, the American College of Medical Genetics (ACMG) consensus indicates that the homozygote for the C677T variant of the MTHFR gene increases the likelihood for hyperhomocysteinemia, which in turn increases the risk of arterial thrombosis. However, this mutation only accounts for a third of hyperhomocysteinemia cases, and the mutation itself is not associated with arterial thrombosis except in conjunction with hyperhomocysteinemia; therefore, the clinical use of MTHFR testing is less informative than plasma homocysteine measurement [22].

One of the limitations of our study was that testing for anti-neuronal antibodies, such as anti-NMDAR, was not performed; autoimmune encephalitis was considered in the differential diagnosis. Given the acute neuropsychiatric presentation and parotid inflammation, antiviral therapy was initiated empirically, despite serological findings limited to anti-CMV IgG. This approach was chosen to address the probability of viral encephalitis or viral reactivation in the context of a severe clinical picture.

In conclusion, the present case highlights the possible relationship between psychiatric and thrombotic disorders, suggesting that both the MTHFR mutation and protein S deficiency could lead to psychotic disorders. Even though MTHFR mutation testing is not part of the routine assessment, since it has a limited predictive value when present by itself, the coexistence of it with other prothrombotic factors, such as protein S deficiency, increases its clinical significance. Therefore, the early detection of thrombotic risk factors in early-onset psychiatric disorders is essential for the comprehensive management of patients. In this context, this paper may contribute novel insights to enhance diagnostic precision and contribute to individualized and effective treatments.

## Figures and Tables

**Table 1:** TORCH profile.

Study	Outcome	Reference Value
Toxo IgG	<5 UI/mL	Non-reactive
Toxo IgM	0.02 L/mL	Non-reactive
Rubella IgM	0.16 IC	Non-reactive
Cytomegalovirus IgG	2.42 IC	Reactive > 1.1
Cytomegalovirus IgM	0.2 IC	Non-reactive
Herpes I and II IgG	0.63 IC	Non-reactive

**Table 2:** Thrombophilic and autoimmune profile. Analysis of genetic and coagulation factors.

Study	Outcome	Reference Value
Factor II mutation 20210 genotype	Not detected	Not detected
Mutation factor V (Leiden mutation)	Not detected	Not detected
MTHFR variant p.Ala222Val	Detected	Not detected
C3 complement	119.620	90–180
C4 complement	26.835	10–40
Anti-SSB Antibodies (LA)	3.3	<20
Anti-NATIVE DNA antibodies (IFI)	Negative	Negative
Antineutrophil cytoplasmic antibodies (ANCA P)	Negative	Negative
Perinuclear neutrophil antibodies	Negative	Negative
Myeloperoxidase	Negative	Negative
Anti-SSA antibodies (RO)	4.9	<20
Anti-beta 2 glycoprotein IGG antibodies	6.4	<20
Anti-beta 2 glycoprotein IGM antibodies	2.1	<20
Lupus anticoagulant	1.14	<1.16
Anticardiolipin IGG antibodies	4.0	<20
Anticardiolipin antibodies IGM	2.1	<20
Coagulation protein C	77.0	65–130
Coagulation protein S	35.0	54.0–103.0
Coagulation factor XII	65.9	36.0–159.0
Prothrombin time	11.8 s	11–13 s
International normalized ratio (INR)	1	0.8–1.2
Activated partial thromboplastin time	23.2 s	25–45 s

## Data Availability

The raw data supporting the conclusions of this article will be made available by the authors upon reasonable request.

## Abbreviations

The following abbreviations are used in this manuscript:

MTHFR	methylenetetrahydrofolate reductases
PS	protein S
PC	protein C
FVL	Factor V Leiden
C4BP	complement protein 4
APC	activated PC
TFPI	tissue factor pathway inhibitor
Ala222Val	alanine to valine
BDNF	brain-derived neurotrophic factor
CSF	cerebrospinal fluid
EEG	electroencephalogram
MRI	magnetic resonance imaging
ATP	adenosine triphosphate
